# Effects of the Hot-Drawing Process on the Pore Parameters, Gas Absorption and Mechanical Performances of Activated Carbon-Loaded Porous Poly(m-Phenylene Isophthalamide) Composite Fibres

**DOI:** 10.3390/polym16243452

**Published:** 2024-12-10

**Authors:** Xiaosong Li, Bo Li, Qibin Xu, Lingcheng Meng, Deyang Wu, Pengqing Liu, Fabien Salaün, Shengchang Zhang

**Affiliations:** 1 College of Polymer Science & Engineering, Sichuan University, Chengdu 610065, China; lxs1231@scu.edu.cn (X.L.); bobleec1@163.com (B.L.); xuqibin2015@163.com (Q.X.); menglingcheng163@163.com (L.M.); liupq@scu.edu.cn (P.L.); 2 Sichuan Develop China Tech New Materials Co., Ltd., Meishan 620000, China; wudeyang@sc-dct.com; 3 ULR 2461–GEMTEX–Génie et Matériaux Textiles, Ecole Nationale Supérieure des Arts et Industries Textiles, Université de Lille, F-59000 Lille, France

**Keywords:** breathable chemical protective clothing, porous PMIA fibres, the exposure-immobilization of activated carbon, pore parameters manipulation, adsorption–desorption behaviour

## Abstract

Poor breathability, inadequate flexibility, bulky wearability, and insufficient gas-adsorption capacity always limit the developments and applications of conventional chemical protective clothing (CPC). To create a lightweight, breathable, and flexible fabric with a high gas-absorption capacity, activated carbon (AC)-loaded poly(m-phenylene isophthalamide) (PMIA) porous composite fibres were fabricated from a mixed wet-spinning process integrated with a solvent-free phase separation process. By manipulating the pore parameters of as-spun composite fibres, the exposure-immobilization of AC particles on the fibre surface can offer a higher gas-absorption capacity and better AC-loading stability. To improve the mechanical properties of AC-loaded porous as-spun fibres and further optimize the pore-locking structures, the impact of the hot-drawing process on the evolution of pore parameters and the corresponding properties (including the gas absorption capacity, the mechanical performance, and the stability of AC particles during loading) was clarified. After the hot-drawing process, the inhomogeneous pore morphologies composed of mesopores/micropores from as-spun fibres changed into homogeneous and decreased mesopores. With the decrease in structural defects in homogeneous morphologies, the tensile strength of AC-loaded PMIA porous-drawn fibres increased to 1.5 cN/dtex. Meanwhile, the greater total pore volume and specific surface area after hot drawing also maintained the gas-absorption capacity of drawn composite fibres at 98.53 mg/g. Furthermore, the AC-loaded PMIA porous composite fibres also showed comparable performance to the commercial FFF02 absorption layer in terms of static absorption behaviour for different gas molecules and absorption–desorption multi-cycling evaluations. In addition, due to the size reduction in mesopores after the hot-drawing process, the loading stability of AC particles in the stretched composite fibres was more substantial.

## 1. Introduction

To effectively address various public health events and chemical accidents, applying chemical protective clothing (CPC) is crucial and indispensable [1,2,3]. The wearing comfort and the CPC’s gas-absorption capacity directly impact the working efficiency and the serving safety of the wearer [4,5]. To date, most commercial CPCs are based on the presence of activated carbon (AC, as adsorbent) coated on the surface of textile structures. These structures are multi-layered, with low breathability, insufficient flexibility due to the use of binders, and poor wear resistance [6]. One way to avoid using binders while improving breathability, CPC flexibility, and reducing weight is to incorporate AC particles directly into the fibres during the spinning process, enabling the development of gas-absorbing textiles based on a multi-layered structural design [1,7,8]. However, the application and development of breathable and flexible CPCs based on AC-filled fibre are limited due to poor gas absorption caused by a random distribution of AC particles in the core of the composite fibres [9,10]. In other cases, the particles are located on the surface of the fibres, but their poor adhesion also leads to a progressive reduction in gas-absorption capacity [11,12].

Inspired by the exposure-immobilization of enzymes on the porous surface of framework materials [13,14,15], one effective strategy for improving the functionality of this type of fibre is to design a surface-functionalized porous fibre with particles partially entrapped in the porous matrix [16,17]. Porous fibres have a high specific contact surface, which increases the possibility of exposure of AC particles to the environment, favouring direct contact between the toxic gas and AC particles and thus improving the efficiency of gas absorption [18]. In addition, the cage effect produced by tiny pores traps AC particles in the polymer matrix, keeping them on the fibre surface. Thus, manipulating the suitable pore parameters of AC-loaded polymeric porous composite fibre became the crucial key to maximizing the exposure of AC particles from large pores and the anchorage-locking of AC particles from small pores before simultaneously enhancing the gas-absorption capacity and the stable AC-loading of AC-loaded composite fibres [19]. Among the various methods for pore-morphology formations and controls, the most widely used method for manufacturing porous polymer fibres is the phase separation method, particularly non-solvent-induced phase separation (NIPS) from the wet spinning process due to its accurate control in pore designs, easy implementation in operations, less energy consumption, and lower cost in large-scale production [20,21,22]. When the polymer spinning solution is introduced into the coagulation bath, diffusion phenomena between solvent and non-solvent molecules, respectively, from the polymer solution into the coagulation bath and vice versa, lead to the occupation of part of the free volumes present in the fibre core by non-solvent molecules [23]. The porous morphology of polymer fibres is obtained after the non-solvent molecules have been removed by post-treatment or dry extraction [24]. Thus, porous morphology can be modulated by controlling the diffusion kinetics of solvent and non-solvent molecules during the spinning process [25,26].

The double diffusion process is generally controlled by adjusting the formulations of the spinning solution and the coagulation bath to the surrounding conditions [27]. For example, during the wet-spinning process of porous composite fibres composed of poly(m-phenylene isophthalamide) (PMIA) as a matrix and AC as functional fillers, their pore morphologies can be tailored effectively by adjusting the compositions and temperatures of coagulation bath based on NIPS technology [28]. For the results, porous structures with sponge-like and mesoporous morphologies offered a better exposure and immobilization effect of AC particles to achieve a 120 mg/g gas-absorption capacity and stable filler loading [29,30]. However, the creation of porous structures and the addition of AC particles will inevitably deteriorate the mechanical performances of surface-functionalized fibre. And the weakening of mechanical properties will greatly limit the subsequent application of porous AC-filled PMIA fibres [31,32,33]. For example, the insufficient strength not only led to the frequent fracture of yarn during weaving and then reduced the stability and the production speed of related textile productions but also increased the damaging risk of CPCs and then threatened the safety of the wearer. Therefore, to further promote the real applications and developments of these porous AC-filled fibres, it is necessary to look for a strategy to improve mechanical properties while keeping the exposure-immobilization effects from impacting well-designed pores. Hot drawing has been considered an effective method for improving the mechanical performance of porous AC-filled composite fibres while enhancing the crystallinity ratio through macromolecular chain orientations [34,35,36]. However, these characteristics are enhanced at the cost of damaging the original well-designed pore morphologies during the hot-drawing process [37,38]. Therefore, to improve the mechanical properties of porous AC-filled polymer fibres while maintaining the exposure-immobilization effects offered by the specific pore morphologies, it is necessary and significant to study the impact of the hot-drawing process on the evolutions of pore morphology, the gas-absorption capacity, AC-loading stability and mechanical properties of AC-filled polymer fibres.

This work used PMIA, a high-performance polymer with excellent chemical corrosion resistance, exceptional thermal stability, and good flame retardancy, as the polymer matrix to manufacture the CA-filled composite fibre for gas absorption [39,40]. PMIA fibres with porous morphologies containing AC particles were prepared from blend wet spinning coupled with the NIPS process and followed by a hot-drawing step with a total draw ratio of 1.2 at 290–295 °C and a heat setting at 300 °C. To clarify the effects of the hot-drawing process on the structures and performance of AC-loaded PMIA composite fibres, the evolutions of pore parameters, gas-absorption capacity, mechanical properties, and the AC-loading stability of composite fibres before and after the hot-drawing process were characterized by N_2_ isotherm adsorption–desorption measurements, mechanical properties, benzene adsorption performance tests, and mechanical friction tests. In addition, to further verify the application value of this AC-filled PMIA composite fibre, comprehensive comparisons with the commercial FFF02 absorption layer were also carried out in terms of static absorption behaviour for different gas molecules and adsorption–desorption capacity based on multi-cycle evaluations. This implementation process enables pore morphologies to be controlled during the NIPS process. The heat-stretch optimization treatment paves the way for a lightweight, breathable, and flexible CPC with a high gas-absorption capacity, which can be used in biochemical accident handling and disaster emergency response.

## 2. Materials and Methods

### 2.1. Raw Materials

A poly(m-phenylene isophthalamide) (PMIA) solution was purchased from Times New Material Technology Co., Ltd. (Zhuzhou, China). Polyethylene glycol (PEG, 2000 g/mol, AR), methanol (AR), benzene (AR), dimethylacetamide (DMAc, AR), and n-hexane (AR) were purchased from Kelong Chemical Co., Ltd. (Chengdu, China). AC particles (HNAC500S) were acquired from the Chemical Defense Research Institute in the Military Academy of Sciences (Beijing, China). The FFF02 protective clothing was obtained from Protective Shield Safety and Security Co., Ltd. (Xi’an, China).

### 2.2. Preparation of the AC-Loaded PMIA Porous As-Spun Fibres

First, the AC particles and DMAc were pre-mixed in a star ball mill to obtain an AC slurry with a mean particle size of approximately 1 μm. Next, the AC slurry was mixed with PMIA/DMAc solution and PEG (molecular weight 2000 g/mol) and mechanically stirred at 70 °C for 6 h to prepare the blend spinning solution with a solid content of 17 wt% (mass ratio among PMIA, AC, PEG, and DMAc was 13.6:3.4:0.72:86.28). A static de-bubbling process was carried out under a vacuum environment at 60 °C for 5 h to remove the bubbles from the mixed spinning solution. Then, a conventional wet-spinning process was carried out to prepare AC-loaded PMIA porous as-spun fibre. Detailed information about the formation process and the operation parameters can be found in our previous publication [28]. This AC-loaded PMIA porous as-spun fibre was named PEG2K-5%.

### 2.3. Hot-Drawing Process of the AC-Loaded PMIA Porous As-Spun Fibres

**Figure 1:**
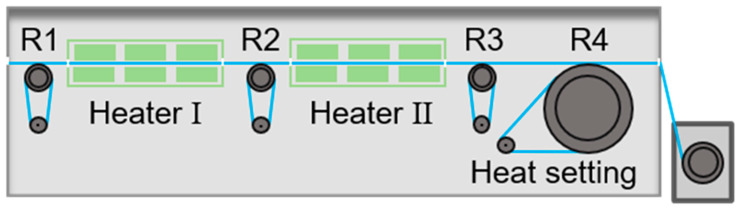
The hot-drawing and the hot-setting processes of AC-loaded PMIA porous as-spun fibre.

As shown in Figure 1, hot-drawing and hot-setting processes were also completed for AC-loaded PMIA porous as-spun fibres. The hot-drawing process was separated into two steps. During the first step, the stretching ratio was set as 1.1 by adjusting the rotation speeds of the roller 1# and the roller 2#, and the drawing temperature was 290 °C. During the second step, the stretching ratio was also set as 1.1 by adjusting the rotation speeds of roller 2# and roller 3#, and the drawing temperature was 295 °C. Meanwhile, the temperatures of roller 1#, roller 2#, and roller 3# were 100 °C, 110 °C, and 120 °C, respectively. Finally, a hot-setting process was carried out at 300 °C for 90 s. And the AC-loaded PMIA porous drawn fibre was labelled PEG2K-5%-p.

### 2.4. Measurements and Characterization

The N_2_ isotherm adsorption–desorption curves of AC-loaded PMIA porous fibres at 77 K were characterized using an automatic physical adsorption analyzer (ASAP 2460, Micromeritics Instruments Corporation, Norcross, GA, USA) to measure the surface area, the pore size distribution, and the pore volume of AC-loaded PMIA porous fibres before and after hot drawing. The de-bubbling temperature was 200 °C. Before the measurements, all the samples were subjected to a pre-treatment at 200 °C for 8 h to eliminate the effect of moisture. The pore size distribution was characterized by analyzing the related desorption curve using the Barret–Joyner–Halenda (BJH) method. The specific surface area was calculated using the Brunauer–Emmet–Teller (BET) method. In contrast, the total pore volume was calculated based on the adsorption isotherm of P/P_0_ = 0.99, and the t-plot method was employed to determine the micropore volume. Finally, the mesopore volume was determined by subtracting the micropore volume from the total pore volume. And the surface morphologies of AC-filled PMIA porous composite fibres before and after the hot-drawing process were also observed by an Inspect F50 field emission scanning electron microscopy (FE-SEM) (Field Electron and Ion Co., Hillsboro, OR, USA). Before the observation, all samples were coated with gold. And the acceleration voltage during the observation was selected as 20 kV.

The porous structure of porous fibres in the cross-section meant that simply using the fracture stress per unit of the cross-section area to characterize the fracture strength (kg/cm^2^) of porous fibre materials was not an entirely accurate reflection of the actual interfacial dimensions of porous fibres nor of the effect of pore structure on their fracture strength. Consequently, utilizing the monofilament breaking force and linear density ratio to characterize the monofilament breaking strength (cN/dtex) of porous fibres was more accurate. A random selection of roots (n) for the prepared AC-loaded PMIA porous fibres was cut to the same length L (mm) using a razor blade. The moisture was then removed in an infrared rapid-drying oven, after which the precise mass m (mg) was measured using a precision torsion balance (JN-B type, Shanghai Second Scales Factory, Shanghai, China). The linear density was calculated by Equation (1). The linear density of AC-filled PMIA as-spun fibre was 6.8 ± 0.2 dtex, and the linear density of drawn composite fibre was 3.1 ± 0.1 dtex.

(1) $$Linear\;Density\;(dtex)=\frac{m\;\times \;10,000}{n\;\times \;L}$$

where n is the fibre number of samples. And L and m are the length and mass of the fibre samples, respectively.

In this study, the breaking force and elongation at the break of fibres were tested using a Fibre Electronic Strength Tester (YG001A type, Jiangsu Taicang Textile Instrument Factory, Taicang, China). The breaking strength can be calculated using Equation (2). The test conditions were set to room temperature, with a fibre pre-tension of 0.2 cN, an upper and lower gripper spacing of 20 mm, and a tensile speed of 20 mm/min. Each group of fibres was measured 20 times, and the resulting average value was recorded.

(2) $$B\mathrm{reaking}\;\mathrm{Strength}\;(\mathrm{cN}/\mathrm{dtex})=\frac{\mathrm{Breaking}\;\mathrm{Force}\;(\mathrm{cN})}{\mathrm{Linear}\;\mathrm{Density}\;(\mathrm{dtex})}$$

**Figure 2:**
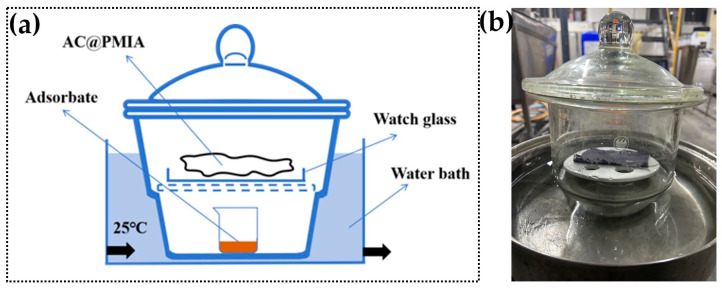
(**a**) The schematic diagram and (**b**) the actual figure of the static adsorption device.

The static adsorption test was conducted using a thermostatic water bath under controlled temperature conditions. Initially, a 250 mL beaker was filled with 100 mL of liquid-adsorbed substances and placed at the bottom of the drying jar. The samples were degassed in a vacuum oven at 90 °C for three hours to remove impurities and moisture. Subsequently, they were placed in a drying tank to absorb volatile adsorbent gasses for 24 h. The longer exposure time ensured that the benzene was completely volatilized and fully adsorbed, as shown in Figure 2.

The quantity of gaseous substances adsorbed per unit mass of the sample, designated as Q (mg/g), can be calculated using Equation (3).

(3) $$Q\;(mg/g)=\frac{m_{1}-m_{0}}{m_{0}}\times 1000$$

where *m*_0_ is the initial mass of the sample. *m*_1_ is the mass of the samples after different static adsorption periods.

Benzene, n-hexane, and methane were used as adsorbed substances for static adsorption at 15 °C, 25 °C, and 35 °C. Measurements were taken at 0.5, 1, 2, 4, 8, 12, and 24 h, respectively. The mass of the samples at each time point was recorded with great precision as m_1_. The adsorbed amount of each substance per unit mass of the sample was then calculated as Q at each time point.

The PEG2K-5%-p and FFF02 adsorbed layer samples, which had been adsorbed for 24 h, were desorbed using an electrically heated blast dryer. The temperature was set at 25 °C and 100 °C, and the weight was recorded at 10 min intervals until no further change in weight was observed, at which point the maximum desorption volume was reached. The samples’ desorption rate (P) was calculated using Equation (4), with *m*_2_ representing the mass of the sample recorded at each designated time point.

(4) $$P=\frac{m_{1}^{\prime }-m_{2}}{m_{1}^{\prime }-m_{0}}\times 100\%$$

where *m*′_1_ is the total weight of the sample when it reaches saturation adsorption. *m*_2_ is the mass of the sample after different static desorption periods, and *m*_0_ is the initial mass of the sample.

**Figure 3:**
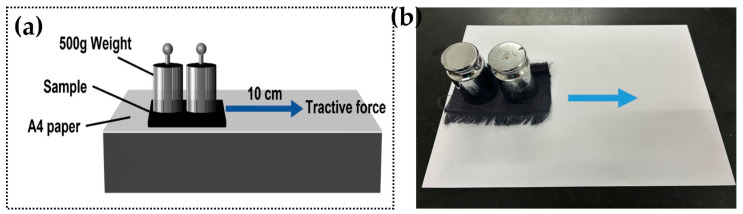
(**a**) The schematic diagram and (**b**) the actual figure of the mechanical friction test.

The length and width of the FFF02 adsorbent layer should be cut, with the activated carbon-loaded side facing inwards. This should be covered with A4 paper and two 500 g weights placed horizontally on the back of the FFF02 sample. The sample was displaced horizontally by 10 cm under the influence of a tensile force, after which it was pulled back to its original position in a direction opposite to that of the initial displacement, as illustrated in Figure 3. Subsequently, the same amount of PEG2K-5%-p porous fibres was spread on A4 paper to perform the same friction experiment. Finally, the friction traces of the FFF02 adsorbent layer and PEG2K-5%-p porous fibre were recorded after 10 rubbing cycles, respectively.

## 3. Results and Discussion

### 3.1. Effects of Hot Drawing on the Pore Morphologies

**Figure 4:**
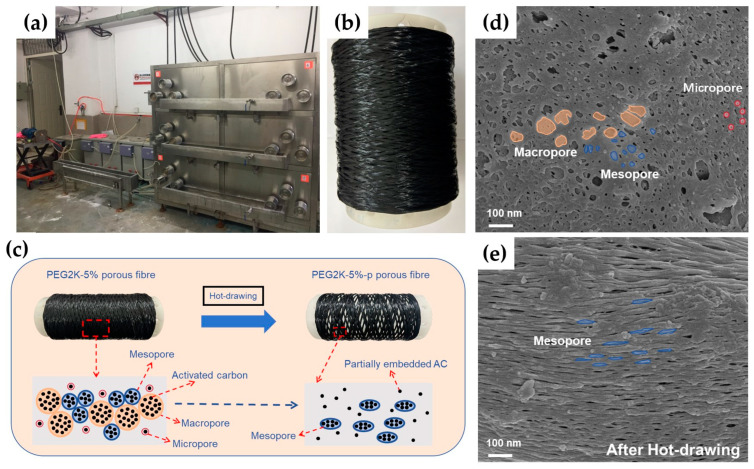
(**a**) Wet-spinning machine used to fabricate AC-filled PMIA porous composite fibre; (**b**) digital image of AC-filled PMIA porous composite fibre; (**c**) the evolution of the pore morphologies of AC-filled PMIA porous composite fibre before and after the hot-drawing process; and (**d**,**e**) the SEM images of the surface morphologies of AC-filled PMIA porous composite fibres before and after the hot-drawing process.

**Figure 5:**
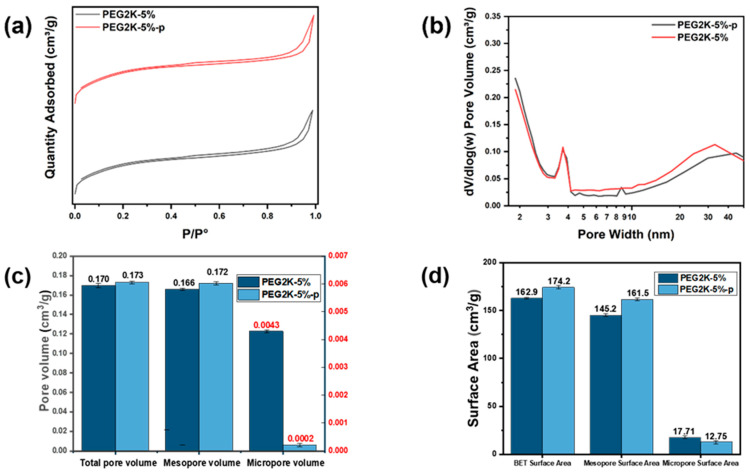
(**a**) N_2_ adsorption–desorption isotherm, (**b**) mesoporous pore size distribution curve, and (**c**,**d**) pore parameters of the PEG2K-5% porous fibres before and after drawing.

The wet-spinning machine used to fabricate AC-filled PMIA porous composite fibres and the digital image of related fibre productions are shown in Figure 4a,b. In order to study the effects of the hot-drawing process on the evolution of pore morphologies, the related SEM images are given in Figure 4d,e. As shown in Figure 4d, before the hot-drawing process, there were macro-pores, mesopores, and micropores distributed on the surface of the AC-filled PMIA porous as-spun fibres. After the hot-drawing process (Figure 4e), the numbers of macro-pores and micropores both decreased significantly. And a large number of elliptical mesopores could be observed on the surface of the AC-filled PMIA porous drawn fibres. Furthermore, the size distribution of these elliptical mesopores exhibited better uniformity. As shown in Figure 4c, during the hot-drawing process, not only were the spherical macro-pores and the spherical micropores stretched into elliptical mesopores, but the original spherical mesopores were also stretched. In the evolution from the micropore or macro-pore to the mesopore, the pore morphology from AC-loaded PMIA porous drawn fibres became more homogeneous. It is consistent with the findings reported in some of the literature [41,42,43]. To further clarify the effects of hot drawing on the pore structure of porous fibres, N_2_ adsorption–desorption tests were performed on PEG2K-5% and PEG2K-5%-p porous fibres. As shown in Figure 5a, the N_2_ isotherm adsorption–desorption curves of PEG2K-5% and PEG2K-5%-p porous fibres could be classified as type IV isotherms. In Figure 5b, on the one hand, the micropore volume (pore size less than 2 nm) from PEG2K-5%-p porous fibres was smaller than the situation from PEG2K-5% porous fibres.

On the other hand, the mesopore volume (pore size ranged from 2 nm to 50 nm) from PEG2K-5%-p porous fibres was more significant than from PEG2K-5% porous fibres. Meanwhile, Figure 5c,d show that the total pore volume and specific surface area from PEG2K-5%-p porous fibres were also higher after the hot-drawing process. The increases in the meso-spore parameters and the decrease in the micropore parameters further verified that the hot-drawing process would transform pore morphology within composite fibre from the micropore to the mesopore. Also, more mesopore morphologies in drawn composite fibre will offer a more specific surface area and higher structural homogeneity.

### 3.2. The Mechanical Properties and Gas-Absorption Capacity

**Figure 6:**
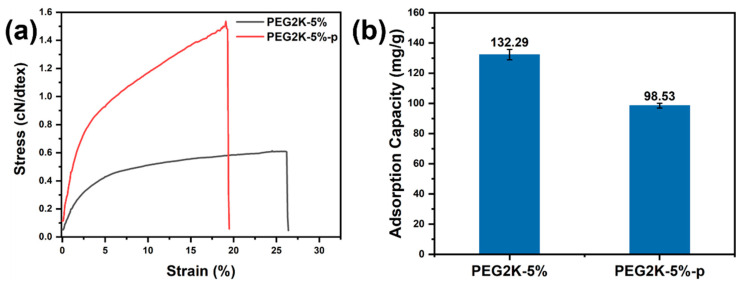
Comparison of (**a**) mechanical properties and (**b**) benzene adsorption properties of PEG2K-5%-p and PEG2K-5%-p.

To clarify the effect of hot drawing on the properties of PEG2K-5% porous fibres, the mechanical properties and benzene adsorption properties of PEG2K-5%-p porous fibres were tested. As shown in Figure 6a, a comparison between PEG2K-5% and PEG2K-5%-p porous fibres revealed a notable enhancement in the monofilament breaking strength of the latter. On the one hand, the improvement in the mechanical properties of the composite fibre could be attributed to the enhancements in the orientation and crystallization of polymeric chains after hot drawing. On the other hand, homogenizing pore morphology after hot drawing also reduced the structural defects and stress-concentrated points within the composite fibre, improving its mechanical properties when faced with an applied stress load. As shown in Figure 6b, the PEG2K-5%-p porous fibre obtained following the drawing exhibited a diminished adsorption capacity for gaseous benzene. This phenomenon might be attributed to the fact that the adsorption of gaseous benzene by the porous fibre was primarily mediated through the micropores and a portion of the mesopores. Additionally, some activated carbon particles within the fibres could be encapsulated during the drawing process, reducing micropores and decreasing benzene adsorption.

Until now, the gas-absorption textiles used for CPCs have mainly depended on AC clothing, which is fabricated from the pre-treatment of precursor textiles (including immersion and polymerization), the pre-oxidation process, the carbonization process, and the activation process [44]. Even though these neat examples of AC clothing present excellent gas-absorption capacity, feelings of stiffness and insufficient toughness make them only capable of serving as a middle layer (absorption layer) to complete the design of multi-layered CPCs. Considering the bulky wearability of multi-layered CPCs and the complex and high-price production process of AC clothing means that there has been a push in AC-filled polymeric composite fibres to improve comfortable wearing (including lightweight, flexible, and breathable), the gas-absorption capacity, and mechanical properties of novel CPCs. Based on the copolymerization process among acrylonitrile (AN), vinylidene chloride (VDC) and AC particles, the acrylonitrile–vinylidene chloride copolymer fibres containing AC particles were prepared [45]. Even though the tensile strength of the acrylonitrile–vinylidene chloride/AC composite fibre can reach 1.3 cN/dtex with 20 wt% AC addition, the absorption capacity for methylene blue only reached about 14 mg/g. The limited absorption capacity was related to the embedding failure of AC particles in the inner composite fibre. For the AC-filled PMIA porous composite fibres, the combination of the exposure-immobilization effects of AC particles on the fibre surface and the hot-drawing enhancement effect of mechanical performance not only improved the gas-absorption capacity of composite fibres to 98.53 mg/g while keeping the stable loading of AC particles but also increased the tensile strength to 1.5 cN/dtex. The existence of AC-filled PMIA porous composite fibres undoubtedly provides a new direction for the development of novel CPCs with lightweight qualities, flexibility, breathability, a high gas-absorption capacity and desired mechanical performance.

### 3.3. Static Adsorption Properties

Activated carbon is a highly nonpolar adsorbent that efficiently adsorbs nonpolar molecules based on the principle of similar compatibility. Additionally, the kinetic diameter of the adsorbed substances is an essential factor affecting the static adsorption curve. For activated carbon with a given pore size distribution, the kinetic diameter of the molecules determines the range of the pore space that can be accessible to the adsorbed substances. Typically, adsorbed substances can only be adsorbed by adsorbents if they are in the influential adsorption pore segments. However, the pore structure of activated carbon is more significant than the kinetic diameter of the adsorbed substances. Therefore, activated carbons with larger kinetic diameters exert a pronounced effect on adsorption within the pores for adsorbed substances. The boiling point of an adsorbed substance exerts a considerable influence on its adsorption onto activated carbon. The magnitude of the boiling point is closely associated with the extent of liquefaction and condensation of the substance. Consequently, the higher the boiling point of organic vapours, the more readily they can condense or liquefy within the microporous space, thereby exhibiting a greater physical adsorption capacity. Methanol, benzene, and hexane are selected as adsorbents based on different polarities, kinetic diameters, and boiling points, and the physical parameters of the three adsorbed substances are listed in Table 1.

**Figure 7:**
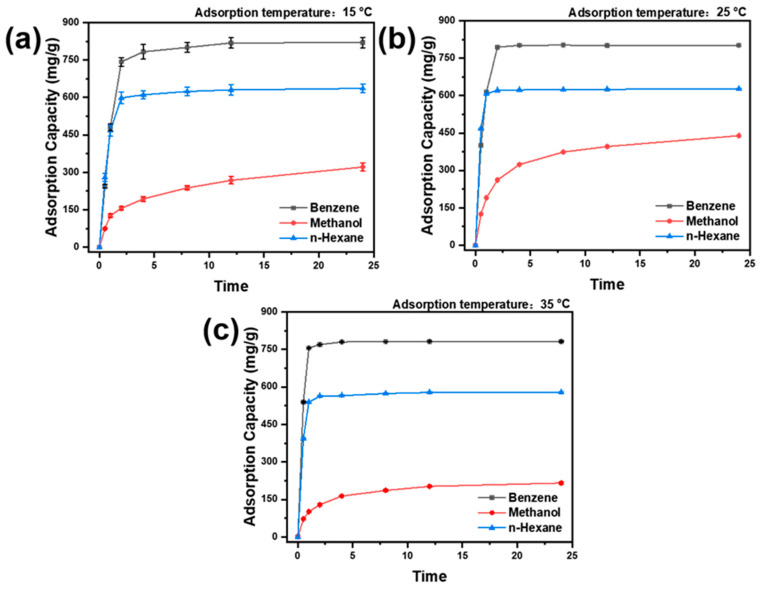
Static adsorption curves of AC powder for different adsorbed substances at various temperatures (**a**): 15 °C; (**b**): 25 °C; and (**c**): 35 °C.

The results of the static adsorption of activated carbon for different adsorbed sub-stances at various temperatures are presented in Figure 7. The AC powder demonstrated a notable adsorption capacity for the three adsorbent gasses. At a temperature of 25 °C, the adsorption of AC powder on benzene and n-hexane reached an equilibrium within approximately four hours, while the adsorption on methanol did not reach equilibrium within 24 h. In terms of adsorption capacity, the total amount of adsorption of AC powder on the three adsorbents for 24 h was benzene (801.62 ± 1.07 mg/g) > n-hexane (627.33 ± 1.02 mg/g) > methanol (439.50 ± 1.03 mg/g), respectively. Regarding the adsorption rate, AC powder exhibited the fastest adsorption rate for n-hexane and the slowest adsorption efficiency for methanol. This was due to methanol’s greater polarity than benzene and n-hexane and its smaller kinetic diameter, resulting in poor methanol adsorption by AC powder. Furthermore, methanol’s boiling point is lower than benzene and hexane’s boiling point, making methanol not easily liquefied or coalesced on AC powder. Additionally, methanol’s saturated vapour pressure was higher than benzene’s vapour pressure, making methanol desorption more efficient. Consequently, the adsorption rate of AC powder on methanol was the slowest, and the adsorption amount was the lowest. In the case of benzene, its kinetic diameter and boiling point were the largest, allowing benzene to be easily adsorbed by AC powder. However, due to its low saturation vapour pressure, benzene was not easily volatilized or desorbed after adsorption. Although benzene had a particular polarity that negatively affected its adsorption by AC powder, the advantages of its kinetic diameter, boiling point, and saturation vapour pressure offset this adverse effect, resulting in the largest adsorption of benzene by AC powder. Concerning n-hexane, its polarity was the lowest, leading to the fastest adsorption rate of n-hexane by AC powder. However, due to its maximum saturated vapour pressure, smaller kinetic diameter, and lower boiling point, AC powder’s total amount of adsorption was not the largest.

Furthermore, methanol, benzene, and n-hexane adsorption curves were examined at varying temperatures of AC powder to elucidate the impact of temperature on the adsorption of the three substances by activated carbon. As shown in Figure 7, the quantity of methanol adsorbed by AC powder exhibited a dual trend as the adsorption temperature increased. Initially, the adsorption rate increased, followed by a decline due to the exothermic nature of the adsorption process, whereby the increase in temperature inhibits the adsorption of AC powder for the three adsorbents while simultaneously increasing its desorption capacity. This leads to a decrease in the total amount of adsorption.

Conversely, the temperature increase enhanced the gas molecules’ movement capacity, facilitating their diffusion into the pores. After 24 h of adsorption at 15 °C, the adsorption of AC powder for methanol was lower than that at 25 °C. This was because methanol gas molecules had a poorer ability to move at 15 °C, resulting in a slower adsorption rate of AC powder and decreased methanol adsorption.

**Figure 8:**
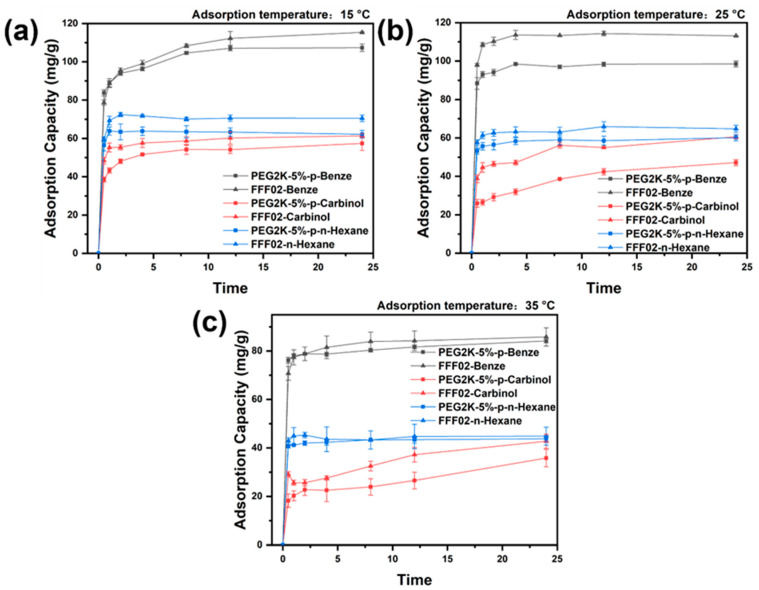
Static adsorption curves of PEG2K-5%-p porous fibre and FFF02 adsorption layer for different adsorbed substances (benzene, methanol, and n-hexane) at various temperatures (**a**): 15 °C; (**b**): 25 °C; and (**c**): 35 °C.

The static adsorption effects of the PEG2K-5%-p porous fibre and the FFF02 adsorption layer on various adsorbed substances at different temperatures are shown in Figure 8 and Table 2. Although the adsorption capacity of the FFF02 adsorption layer for the three adsorbed substances was higher than that of PEG2K-5%-p, the difference was insignificant, only within 15 mg/g. It shows that the PEG2K-5%-p porous fibre is expected to have a good effect when applied to the adsorption layer of chemical protective clothing. When the adsorption temperature is 25 °C, the adsorption rate of the FFF02 adsorption layer for the three adsorbed substances is higher than that of the PEG2K-5%-p porous fibre, which may be because the FFF02 adsorption layer adheres to activated carbon on the fabric surface via the binder. In contrast, the activated carbon in the PEG2K-5%-p porous fibre is coated inside the fibre.

In adsorption, the activated carbon in the FFF02 adsorption layer can adsorb the substance almost without hindrance. In contrast, the adsorption process of the PEG2K-5%-p porous fibre must occur through a series of complex pores. In addition to the activated carbon in the PEG2K-5%-p porous fibres, mesopores on the porous fibres also adsorb adsorbents through capillary condensation. Capillary condensation causes the adsorbent to condense in the porous fibre. Compared with the strong adsorption of activated carbon, the adsorption capacity of the porous fibre is lower, and the adsorption rate of the porous fibre is also reduced to some extent. The adsorption rates of the PEG2K-5%-p porous fibre and the FFF02 adsorption layer displayed similar patterns at different temperatures, and both increased with temperature increases.

### 3.4. Static Desorption Properties

**Figure 9:**
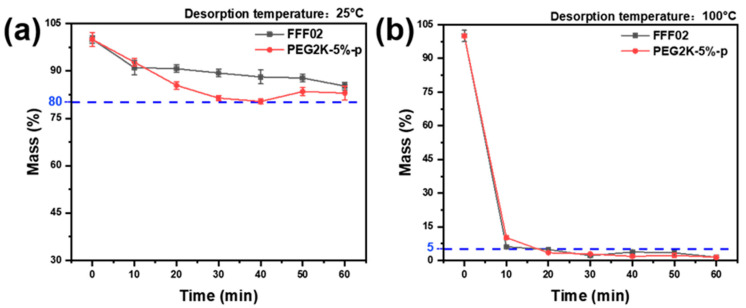
Desorption curves of the FFF02 adsorption layer and PEG2K-5%-p porous fibres at (**a**) 25 °C and (**b**) 100 °C, respectively.

The FFF02 adsorbed layer and PEG2K-5%-p porous fibre were desorbed in a blower drying oven at 25 °C and 100 °C, respectively, following 24 h of benzene adsorption at 25 °C. The desorption curves are shown in Figure 9.

As shown in Figure 9a, the desorption rate of the FFF02 adsorbent layer was slightly lower than that of the PEG2K-5%-p porous fibre at 25 °C. However, both exhibited a desorption rate of less than 20%. The discrepancy in performance might be attributed to the differing mechanisms of benzene adsorption by the two materials. The PEG2K-5%-p porous fibre exhibited dual adsorption capacity, utilizing the pore structure of the porous fibre and AC for benzene capture. In contrast, the FFF02 adsorbent layer demonstrated predominantly AC-mediated benzene adsorption, with the adsorption stability of AC on benzene exceeding that of benzene adsorption by the pore structure of the porous fibre. Figure 9b shows that the desorption rate of the FFF02 adsorbent layer and the PEG2K-5%-p porous fibre was greater than 95%, following desorption at 100 °C for 30 min. It indicated that the adsorption of benzene by the FFF02 adsorbent layer and the PEG2K-5%-p porous fibre was a physically adsorptive process, whereby the desorption of benzene occurred under the condition of a temperature increase.

### 3.5. Resorption Properties

**Figure 10:**
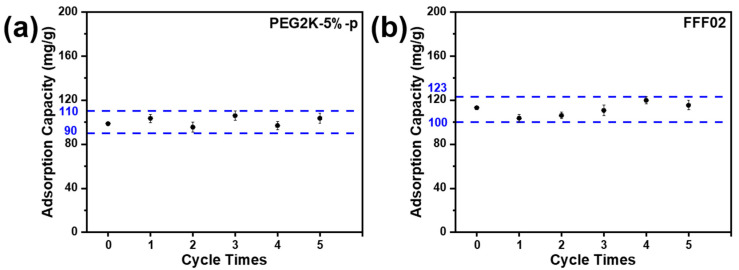
Re-adsorption capacity benzene by (**a**) FFF02 adsorption layer and (**b**) PEG2K-5%-p po-rous fibre at 25 °C, respectively.

To clarify the effect of the static thermal desorption process on the re-adsorption performance of the samples, the PEG2K-5%-p porous fibre and the FFF02 adsorbent layer, which underwent the static thermal desorption treatment, were tested for multiple re-adsorption cycles of benzene at 25 °C. The amount of the adsorbed benzene after 24 h of various re-adsorption cycles of the samples was obtained (Figure 10). The benzene adsorption amounts of the PEG2K-5%-p porous fibre after five adsorption–desorption cycles ranged from 90 to 110 mg/g. At the same time, the benzene adsorption amounts of the FFF02 adsorbent layer after the same number of cycles ranged from 100 to 123 mg/g. The results demonstrated that static thermal desorption had no impact on the re-adsorption performance of the samples, indicating that both the PEG2K-5%-p porous fibre and the FFF02 adsorbent layer were suitable for reuse.

### 3.6. AC-Loaded Stability

**Figure 11:**
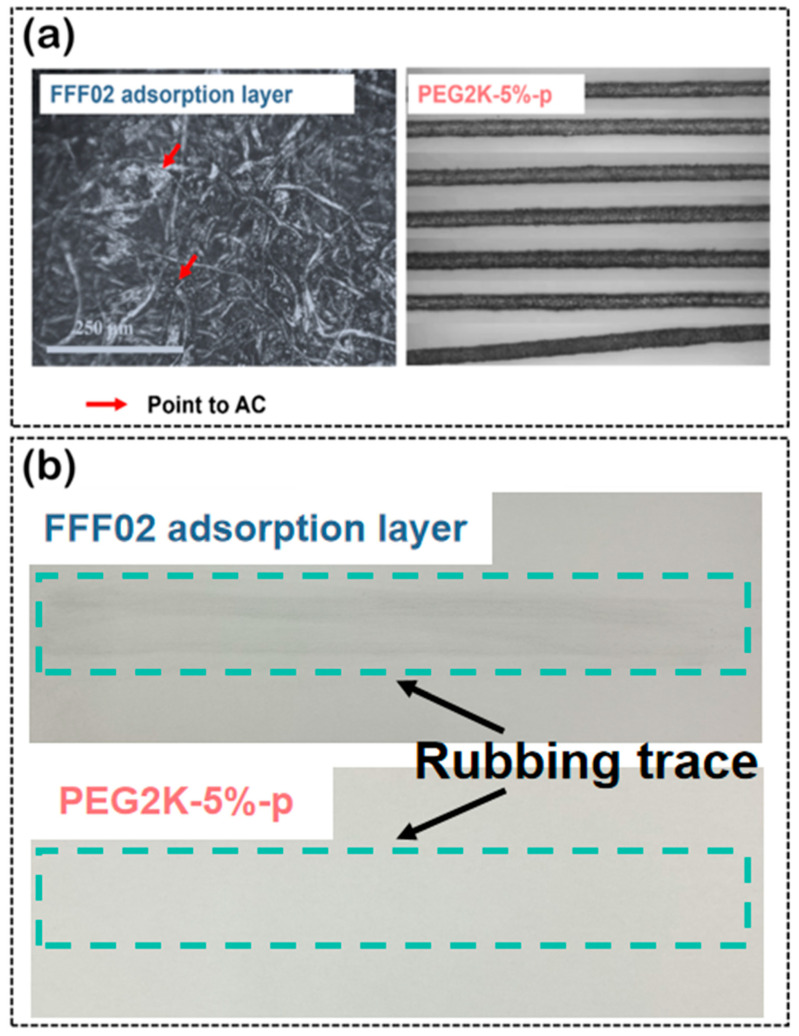
(**a**) The images of the FFF02 adsorption layer and the PEG2K-5%-p porous fibres; (**b**) the friction results of the FFF02 adsorption layer and the PEG2K-5%-p porous fibres.

The morphology of the FFF02 adsorbent layer and the PEG2K-5%-p porous fibres under the optical microscope is shown in Figure 11a. The activated carbon was distributed on the surface of the FFF02 adsorption layer or in the crevices of the fabric. In contrast, only a small amount of AC particles could be observed on the surface of the PEG2K-5%-p porous fibre. As shown in Figure 11b, even though the FFF02 adsorbent layer and the PEG2K-5%-p porous fibre both left friction traces on the A4 paper after 10 cycles of friction, the rubbing trace from the FFF02 adsorbent layer was more apparent compared to that from the PEG2K-5%-p porous fibre. For the FFF02 adsorbent layer, due to the poor adhesion between the AC particles and the textile surface, AC particles easily fell from the textile surface after mechanical friction. For the PEG2K-5%-p porous fibre, the pore-locking effect of the porous fibre limited the movement of exposed AC particles, preventing their fall from the surface of the composite fibre. Therefore, the AC particles within the PMIA porous composite fibre expressed better loading stability.

## 4. Conclusions

A heat-stretching step produces chain orientation due to macromolecular chain reorganization. It affects the degree of crystallinity and, therefore, the mechanical properties of PEG2K-5% porous fibres. In addition, the melt-drawing process also promoted a change in the pore morphologies that existed in porous AC-filled PMIA composite fibres, from inhomogeneous morphologies composed of mesopores/micropores to homogeneous morphologies comprising more mesopores only, increasing in total pore volume and the specific surface area. As a result, the PEG2K-5%-p porous fibres obtained by the melt-drawing process also exhibited excellent gas-absorption capacity for the various adsorbed substances, comparable to the performance of the commercial FFF02 adsorption layer. Porous PEG2K-5%-p fibres and FFF02 adsorbed layers showed good adsorption stability after 24 h of adsorption at 25 °C, with the ability to be almost wholly desorbed at 100 °C. The rate of desorption also increased with temperature. The results of multiple adsorption–desorption cycles also showed that PEG2K-5%-p porous fibres exhibit good stability in their adsorption capacities after long-duration cycles. Combined with the excellent loading stability of AC particles in the composite fibre, PEG2K-5%-p porous fibres showed great potential for use as a structural adsorption component to design breathable, flexible, and lightweight chemical protective clothing with a high gas-absorption capacity. However, during the hot-drawing process, the existence of porous structures and a lot of AC particles at the stress concentration point and the structural defects still depressed the actual hot-drawing ratio and the stretching speed of AC-filled PMIA as-spun fibre. Therefore, the improvement in the mechanical properties of AC-filled PMIA composite fibre after hot drawing is relatively limited. Better methods for further enhancing the stretchability of AC-filled PMIA porous as-spun fibres during the hot-drawing process will be expected in the future.

## Figures and Tables

**Table 1:** Physical properties of three adsorbed substances.

Adsorbates	Kinetic Diameter (nm)	Polarity	Boiling Point (°C)	Saturated Vapour Pressure at 20 °C (kPa)
Methanol	0.36	6.00	64.7	12.97
Benzene	0.58	3.30	80.0	10.03
n-Hexane	0.43	0.06	69.0	16.16

**Table 2:** Total static adsorption amount of the FFF02 adsorption layer and PEG2K-5%-p porous fibre for different adsorbates at different temperatures (24 h).

Samples	Temperatures	Total Static Adsorption Amount (mg/g)		
		Methanol	Benzene	n-Hexane
PEG2K-5%-p	15 °C	57.40 ± 3.66	107.35 ± 1.95	62.18 ± 0.96
	25 °C	47.12 ± 1.60	98.53 ± 1.58	60.13 ± 1.60
	35 °C	35.83 ± 3.60	84.13 ± 0.73	43.79 ± 1.28
FFF02	15 °C	61.35 ± 0.98	115.36 ± 0.40	70.50 ± 1.70
	25 °C	60.64 ± 1.04	113.14 ± 0.14	64.80 ± 1.81
	35 °C	42.74 ± 2.91	85.85 ± 3.77	44.85 ± 3.71

## Data Availability

Data are contained within the article.
